# The Moderated Mediating Effects of Social Media Identity and Loneliness on the Relationship Between Problematic Internet Use and Mental Health in China: Nationwide Cross-Sectional Questionnaire Study

**DOI:** 10.2196/57907

**Published:** 2025-02-26

**Authors:** Chenxi Liu, Yushu Liu, Chaojie Liu, Rujiao Lin, Xi Wang, Xinyi Zhang, Yibo Wu, Dan Wang

**Affiliations:** 1 School of Medicine and Health Management Tongji Medical School Huazhong University of Science and Technology Wuhan China; 2 School of Psychology and Public Health La Trobe University Melbourne Australia; 3 School of Public Health Peking University Beijing China; 4 School of Management Hubei University of Chinese Medicine Wuhan China; 5 Hubei Shizhen Laboratory Hubei University of Chinese Medicine Wuhan China; 6 Research Center for Traditional Chinese Medicine Development Hubei University of Chinese Medicine Wuhan China

**Keywords:** problematic internet use, loneliness, anxiety, depression, mediation and moderation model

## Abstract

**Background:**

Mental health disorders are a major public health challenge, and problematic internet use (PIU) may play an important role in this issue. However, the underlying mechanisms of PIU and its effects on mental health have not yet been elucidated.

**Objective:**

This study examines the mediating effect of loneliness on the relationship between PIU and mental health, as well as the moderating effect of social media identity on the relationships among PIU, loneliness, and mental health.

**Methods:**

A cross-sectional questionnaire survey was conducted on 21,292 participants recruited using a multistage stratified sampling strategy from 31 provinces/regions in mainland China from June 20 to August 31, 2022. This study assesses PIU (PIU questionnaire-short form-6), depression (9-item Patient Health Questionnaire [PHQ-9]), anxiety (7-item Generalized Anxiety Disorder scale [GAD-7]), loneliness (3-item loneliness scale), and social media identity (identity bubble reinforcement scale). Additionally, we collected the sociodemographic characteristics of the participants. Participants whose total score of PHQ-9≥15 or total score of GAD-7≥10 were considered to have moderate or severe symptoms of depression or anxiety, respectively. A moderated mediation model was established to examine the mediating effect of loneliness on the association between PIU and mental health outcomes (depression and anxiety), moderated by social media identity.

**Results:**

Approximately 22.80% (4854/21,292) and 14.20% (3023/21,292) of the respondents reported moderate or severe symptoms of depression and anxiety, respectively. Loneliness significantly mediated the association between PIU and mental health outcomes, explaining 42.53% and 45.48% of the total effect of PIU on depression and anxiety, respectively. Social media identity significantly moderated the associations between PIU and depression (β=0.002, 95% CI 0.001-0.002), PIU and anxiety (β=0.001, 95% CI 0.000-0.002), loneliness and depression (β=0.010, 95% CI 0.007-0.013), and loneliness and anxiety (β=0.007, 95% CI 0.004-0.010), but not between PIU and loneliness (β=0.000, 95% CI –0.003 to 0.003). Higher levels of social media identity were significantly associated with lower levels of loneliness (β=–0.018, 95% CI –0.020 to –0.016).

**Conclusions:**

Addressing loneliness may serve as a valuable approach to mitigate the impact of PIU on mental health outcomes. However, social media identity poses a significant challenge in addressing health issues linked to PIU.

## Introduction

### Background

Mental health disorders, which are significant contributors to the global burden of disease, are a major public health challenge worldwide. Over the past 3 decades, its burden, measured in disability-adjusted life-years, has increased from 80.8 million in 1990 to 125.3 million in 2019, with no indication of a decrease in the near future [1]. The COVID-19 pandemic has further exacerbated mental health problems, resulting in an additional 53.2 million cases of depression and 76.2 million cases of anxiety—the 2 most prevalent mental health disorders [2]. Responding to the prolonged mental health impacts of COVID-19 has proven challenging because health care systems are ill-prepared [3].

Mental health disorders are influenced by a multitude of factors [4], with complex interactions among biological, psychological, social, cultural, and other elements [5]. The rapid advancement of technology, particularly the widespread use of the internet, has garnered significant attention for its impact on mental health and vice versa [6]. Existing studies [7] have shown that there is a potential bidirectional relationship between internet use and mental health disorders, in which internet overuse may induce mental health disorders, for example, depression and anxiety, which could in turn enhance one’s addiction to the internet. Although the relationships between internet use and mental health disorders remain unclear and different studies have argued different directional relationships between them, it is undeniable that the internet has profoundly transformed human society, altering how people communicate and interact, reducing communication barriers, lowering costs, and promoting interpersonal bonds and social networks [8].

In this study, we focused on the impact of internet use on mental health disorders, and existing studies have shown that maladaptive or excessive internet use has been linked to adverse effects on mental health [9-11]. For example, empirical evidence has found that problematic internet use (PIU) and internet addiction [12-16] increase the likelihood of depression, anxiety, social isolation, and even suicide [7,17-22]. However, the reasons for the associations between PIU and mental health remain unclear. Recent systematic reviews have highlighted several limitations in existing studies, such as small sample sizes, variations in study settings, and lack of moderating or mediating analyses [23,24]. One assumption regarding the link between PIU and mental health disorders is that PIU is inherently connected to loneliness [25]. Although loneliness is not a specific mental health condition, it is associated with major mental health disorders, including depression and anxiety. The effect of internet use on loneliness remains a subject of debate. Some researchers contend that internet use can exacerbate loneliness by compromising offline human interactions [26,27], whereas others argue that it can enhance existing offline relationships and foster new ones [28]. Nonetheless, the association between PIU and loneliness is likely to be bidirectional: PIU can be both a cause (resulting in higher levels of loneliness) and a consequence of loneliness [25].

What has been missing in these discussions is a consideration of individual behaviors: why and how individuals use the internet. Social media has created a virtual space for people to interact, but the reasons people use social media vary significantly. Online platforms can serve as tools for sharing information and exchanging social support in the real world [29,30]. This underscores the importance of the concept of social media identity [31,32]. According to the identity bubble reinforcement model [33], online interactions can lead to the formation of social networks (social identities) where like-minded individuals (homophily) interact with one another, often relying on biased information. Social media identity measures how people perceive their relationships within online networks, with a higher level of social media identity indicating greater involvement with online social networks [34]. It is reasonable to assume that high levels of social media identity may reinforce a sense of belonging to virtual communities at the expense of real-world connections [31]. This, in turn, could affect the association between PIU and loneliness, as well as the impact of PIU on mental health [35,36]. Although previous research has explored the effects of social media identity on online gaming and cyberbullying [35,37], there is a noticeable gap in the literature regarding the role of social media identity in the context of PIU, loneliness, and mental health. Notably, this study was conducted in China, as Asian countries have reported a higher prevalence of PIU, ranging from 20% to 47%, than other regions [38,39].

To better understand the underlying reasons for the associations between PIU and mental health, this study aims to examine the mediating effect of loneliness on the relationship between PIU and mental health, as well as the moderating effect of social media identity on the relationships among PIU, loneliness, and mental health.

### Theoretical Hypotheses

**Figure 1:**
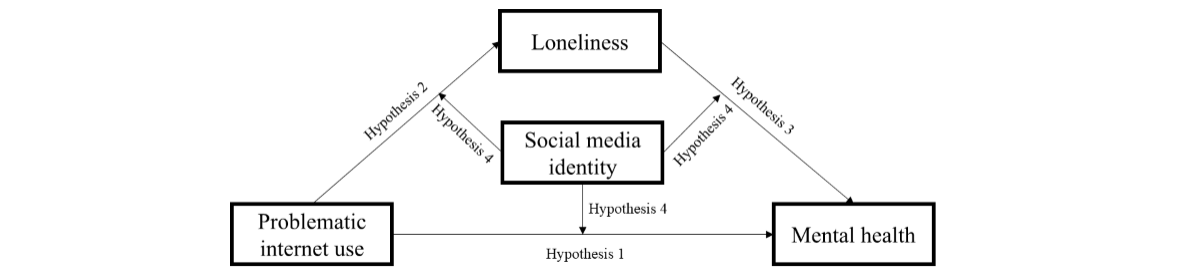
The hypothesized moderated mediation model.

In this study, we tested a moderated mediation model based on 4 hypotheses, examining the interplay among PIU, loneliness, social media identity, and their effects on mental health (Figure 1).

#### Loneliness Mediates the Effects of PIU on Mental Health Disorders

##### Hypothesis 1: PIU Is Positively Associated With Mental Health Disorders

The link between PIU and mental health has been extensively documented [17-20,40]. A recent systematic review revealed a moderate level of evidence supporting a positive association between PIU and depressive symptoms [18]. In general, PIU is associated with withdrawal from real-world social interactions [41]. Excessive internet use can also result in sleep deprivation, unrealistic expectations, and an exaggerated sense of failure [41,42].

##### Hypothesis 2: PIU Is Positively Associated With Loneliness

PIU can lead to displacement, a phenomenon in which internet-based, low-quality relationships replace offline social contacts, even though the internet can strengthen an individual's connectedness with others and expand their social networks [25]. Recent reviews have indicated moderately low levels of evidence supporting PIU as a risk factor for loneliness [25]. Several longitudinal studies have shown that PIU is more likely to be a cause rather than a consequence of loneliness [43-45]. Excessive internet use can lead individuals to withdraw from real-world interactions, intensifying their perception of the absence of companionship [25,46].

##### Hypothesis 3: Loneliness Is Positively Associated With Mental Health Disorders

Evidence has consistently shown that loneliness is a risk factor for mental health disorders, particularly depression and anxiety. Loneliness has negative effects on the social, cognitive, and biological aspects [47,48]. Individuals experiencing loneliness often have less cognitive stimulation, reduced social interactions, and impaired immune functions, all of which are associated with an increased likelihood of mental health disorders [47,48]. This connection between loneliness and mental health disorders has been demonstrated in 2 large longitudinal studies conducted in the United Kingdom and the Netherlands [47,49,50]. Thus, from the evidence in the literature, we inferred that the effect of PIU on mental health disorders is mediated by loneliness, that is, PIU affects loneliness and ultimately translates into mental health disorders.

##### Hypothesis 4: Social Media Identity Moderates the Effects of PIU on Loneliness and Mental Health Disorders

Studies have shown that the extent to which PIU can translate into loneliness and mental health disorders depends on how individuals use the internet [18]. In the identity bubble reinforcement model, social media identity measures how people perceive their relationships with online networks, with a higher level of social media identity indicating greater involvement in and belonging to online social networks [33]. On the one hand, people highly involved in online social networks are more likely to express themselves, receive social support, feel a sense of belonging, be less lonely, and protect themselves from translating PIU and loneliness into mental health problems [30,51,52]. This effect has also been confirmed in risky online behaviors such as gambling [35]. In contrast, in the case of people with PIU but low involvement in and belonging to online social networks, it is expected that their loneliness will be exacerbated and that PIU and loneliness will be translated into mental health disorders. Thus, it is speculated that various levels of social media identity have different impacts on the relationships between individuals’ internet use and loneliness and mental health disorders. Therefore, social media identity was hypothesized to moderate the effects of PIU, loneliness, and mental health in this study, with higher (lower) social media identity indicating a lower (higher) effect of PIU on loneliness and less (greater) likelihood of PIU and loneliness translating into mental health disorders.

## Methods

### Study Design and Participants

The data for this study were extracted from the 2022 nationwide cross-sectional survey, known as the Psychology and Behavior Investigation of Chinese Residents (PBICR-2022). The sample size was determined based on the following formula: n = *Z*^2^_α_ × p × (1 – p) / δ^2^. Based on the prevalence of depression (p) in China as 24% [53], type I error (α) as .05, and acceptable error (δ) as 0.02, the minimum sample size was estimated as 1752. To generate a nationally representative sample, the survey adopted multistage sampling, covering 31 provinces in China (22 provinces, 5 autonomous regions, 4 municipalities). In each province, the planned minimum sample size was determined as 500-2500 according to the proportions of the respective populations to the overall population in China. Finally, the sample size was estimated at 20,000, which is far more than the minimum sample size (n=1752). To select study participants, we first included capital cities from 31 provinces across China. Additionally, 2-12 cities were selected from each noncapital prefecture within each province, resulting in 148 cities. For the 148 municipalities sampled, we adopted random sampling, and the number of communities sampled was determined on the basis of the populations within the sampled municipalities and the ratio of urban to rural communities, resulting in a total of 780 communities. Within each community, the allocation of quotas considered the demographic distributions recorded in the seventh national census, considering both age and sex. Details about the protocol of the PBICR-2022 survey have been published elsewhere [54].

The inclusion criteria for participation in the survey included individuals (1) aged 12 years or older, (2) who had resided in the sampled communities and had not been absent for more than one month within the past year, and (3) who were able to complete the questionnaire independently or with the help of a researcher. We excluded participants who (1) were cognitively impaired in understanding the questionnaire items, (2) had never used the internet or social media, or (3) were unwilling to participate in or were participating in other similar studies during the survey period.

Data collection occurred between June 20 and August 31, 2022, when China was still implementing a zero COVID-19 policy. Household visits were performed by trained researchers whenever local policies permitted such visits. Otherwise, online meetings, typically using platforms such as WeChat or Tencent, were conducted with each participant. The respondents were asked to respond through the Wenjuanxing questionnaire platform. Written informed consent was obtained prior to each survey.

A total of 21,916 responses were returned, and invalid responses (incomplete and contradictory answers or survey times of less than 4 minutes) were excluded. This resulted in a total of 97.15% (21,292/21,916) valid responses. The average age of the included participants was 39.15 (SD 18.64; range 12-100) years.

### Measurements

#### PIU Assessment

PIU was assessed using the 6-item PIU-short form questionnaire. This questionnaire evaluates individual obsessions, neglect, and control behaviors related to internet usage. For example, respondents were asked about their feelings of tension, irritation, or stress when unable to use the internet as long as they wished, their preference for spending time online over sleep, and their attempts to conceal the amount of time spent online [55,56]. The participants were required to rate each item on a 5-point Likert scale, ranging from 0 (never) to 4 (always). A summed score within the range of 0-24 was calculated, with a higher score indicating a greater risk of PIU. The 6-item PIU-short form questionnaire has demonstrated good reliability and validity in several countries [55,56], and its structural validity (via confirmatory factor analysis) and internal consistency (Cronbach α=0.932) have been confirmed.

#### Loneliness Assessment

Loneliness was assessed using the 3-Item Loneliness Scale, which evaluates relational connectedness, social connectedness, and self-perceived isolation [57]. Each item was rated on a 3-point scale, with 0 indicating “hardly ever,” 1 indicating “some of the time,” and 2 indicating “often.” A summed score within the range of 0-6 was calculated, where a higher score indicated greater loneliness. The 3-Item Loneliness Scale has been reported to have good reliability and validity in previous studies [58,59] and in this study, with a Cronbach α coefficient of 0.862.

#### Social Media Identity Assessment

Social media identity was assessed using the Identity Bubble Reinforcement Scale [31]. The Identity Bubble Reinforcement Scale evaluates the extent to which individuals relate to online social networks and consists of 6 items. These items measure social identification (eg, in social media, I belong to a community or communities that I'm proud of), homophily (eg, in social media, I prefer interacting with people who are like me), and information bias (eg, in social media, I feel that people think like me) [31]. The respondents were required to rate each item on a scale ranging from 0 (does not describe me at all) to 9 (describes me completely). A summed score within the range of 0-54 was calculated, with a higher score indicating a higher level of social media identity involvement. The Identity Bubble Reinforcement Scale has shown good reliability and validity in various countries [31], and in this study, it yielded a Cronbach α of 0.924.

#### Mental Health Disorder Assessment

The PBICR-2022 survey included assessments of depression and anxiety, which are among the most common mental health disorders. The 9-item Patient Health Questionnaire (PHQ-9) was utilized to measure depression, while the 7-item Generalized Anxiety Disorder (GAD-7) scale was adopted for assessing anxiety. The respondents were asked to rate their experiences over the past 2 weeks by using a 4-point scale, with responses ranging from 0 (not at all) to 3 (nearly every day). Sample questions included, “How often do I have trouble concentrating on things?” for depression [60] and “How often do I feel afraid that something awful might happen?” for anxiety [61]. A summed score for depression, ranging from 0 to 27, and for anxiety, ranging from 0 to 21, was calculated. A higher score indicates a higher level of symptoms. In this study, participants whose total score of PHQ-9≥15 or total score of GAD-7≥10 were considered as having moderate or severe symptoms of depression or anxiety, respectively. PHQ-9 and the GAD-7 have been widely used in various settings and have been validated in Chinese populations [62,63]. The Cronbach α coefficients of the 2 measurements were 0.919 (PHQ-9) and 0.941 (GAD-7) in our study.

#### Covariates

Previous studies have identified numerous socioeconomic and demographic factors associated with mental health disorders [64-66]. For these factors, data regarding age group (<18, ≥18 to <30, ≥30 to <40, ≥40 to <50, ≥60 to <70 and ≥70 years), sex (male, female), educational attainment (no formal education, primary school, etc), employment (full-time students, employed, freelancers, etc), marital status (unmarried, married, divorced/widowed), household income (≤¥1000, >¥1000 to ≤¥2000, etc; US $1=¥7.31), chronic conditions (yes, no), living area (rural, urban), and region were collected via self-reports. The regions in China were categorized as northern (Beijing, Tianjin, etc), northeast (Liaoning, Jilin, Heilongjiang), northwest (Gansu, Qinghai, etc), eastern (Shanghai, Jiangsu, etc), central (Henan, Hubei, Hunan), southern (Guangdong, Guangxi, Hainan), and southwest (Chongqing, Sichuan, etc) according to the classification of regional economic collaboration among provinces in China [67].

### Statistical Analysis

The characteristics of the study participants were described through frequency distributions for categorical variables and means (medians) and standard deviations (interquartile ranges) for continuous variables. We also examined the normality of the total scores for PIU, loneliness, social media identity, and mental health disorders (depression and anxiety) via Shapiro-Francia tests for normality. The results indicated that all the variables were not normally distributed (*P*<.001). Thus, Spearman correlations were examined among those measurements, and Wilcoxon rank sum tests and Kruskal-Wallis tests were performed to assess differences in PIU, loneliness, social media identity, and mental health disorders among participants with different socioeconomic characteristics.

We employed multivariate linear regression models to test our hypotheses. For the moderated mediation analyses, we utilized the PROCESS macro developed by Hayes [68]. The analysis proceeded in 2 steps. First, we tested the mediating effect of loneliness on the associations between PIU and depression and anxiety disorders. Next, we added the moderating effect of social media identity on different paths within the mediation model. To estimate the indirect effects, we used 5000 bootstrap estimates, which provided a 95% bias-corrected CI with robustness to nonnormally distributed data. Additionally, we calculated the conditional effects of social media identity by examining the associations of all paths between PIU and mental health when the social media identity score was fixed at 1 SD above and below the mean [68].

To assess model robustness and identify potential model heterogeneity, we conducted subgroup analyses on the basis of sex and age, in which mediation and moderated mediation models were reconstructed and tested in a particular subgroup of the population (eg, male participants). As the cross-sectional data did not allow us to establish the temporal precedence of variables, we also examined a model in which PIU served as a mediator for the association between loneliness and mental health disorders. We performed Harman's one-factor analysis to address the potential threat of common method variance in cross-sectional behavioral research. The results indicated that the first common factor in our data explained only 27.99% of the variance, which is below the threshold of 40%. This suggests a low risk of common method variance [69].

The data were analyzed using SPSS 26.0 (IBM Corp). A 2-tailed *P* value <.05 was considered statistically significant. The indirect effects of the mediation and moderation variables were considered statistically significant when zero was not included between the lower and upper bounds of the 95% bias-corrected bootstrap CIs.

### Ethics Approval

This study was approved by the Shaanxi Institute of International Trade and Commerce (JKWH-2022-02). Written informed consent was obtained from participants prior to the survey (for participants aged 12-17 years, approval from their caregivers were also prerequisite for enrollment), and they were provided with the opportunity to opt out at any time during and after the survey. No personal identification information was collected, and all the data were anonymized. There was no compensation for the participants in our study survey.

## Results

### Characteristics of the Study Participants

Approximately half (10,596/21,292, 49.77%) of the respondents were men, whereas 18.1% (3857/21,292) were older than 60 years. Fewer than one-third (6462/21,292, 30.35%) of the respondents resided in rural communities. Most respondents had completed senior high school or above (14,651/21,292, 69%), with an average household income of less than ¥5000 (equivalent to approximately US $684), accounting for 63.02% (13,418/21,292) (Table S1 of Multimedia Appendix 1).

### PIU, Loneliness, Social Media Identity, and Mental Health Outcomes

The respondents had a mean score of 6.49 (SD 5.51) for depression symptoms and 4.75 (SD 4.62) for anxiety symptoms. Compared with male respondents, female respondents (*P*=.003) reported more anxiety problems, while there was no sex difference in depression symptoms (*P*=.07). Respondents with the highest scores for both depression and anxiety (Table S2 of Multimedia Appendix 1) were in the 18-29 years age groups (*P*<.001), unmarried (*P*<.001), had completed postgraduate education (*P*<.001), were engaged in full-time studies (*P*<.001), had a household income of less than ¥1000 per month (equivalent to approximately US $136.80; *P*<.001), resided in rural areas (*P*<.001) and the northeastern region (*P*<.001), and had chronic conditions (*P*<.001).

The loneliness scores were relatively low, with a mean value of 1.57 (SD 1.61) out of a maximum score of 6 (Table 1). In general, respondents with the highest anxiety and depression scores tended to also have higher loneliness scores (Table S2 of Multimedia Appendix 1). The average PIU score was 5.80 (SD 5.46) out of a maximum of 24, indicating a moderate level of PIU. The respondents reported a moderate level of involvement in social media identity, with a mean score of 31.12 (SD 10.99) out of a maximum of 54 (Table 1). The highest levels of PIU and social media identity were found among respondents aged between 18 and 29 years (*P*<.001), those who were unmarried (*P*<.001), had completed postgraduate education (*P*<.001), were engaged in full-time studies (*P*<.001), had an average household income of >¥6000 (equivalent to approximately US $820.80) per month (*P*<.001), lived in urban areas (*P*=.009 for PIU and *P*<.001 for social media identity), and had no chronic conditions (*P*<.001). The respondents residing in the northeast region presented the highest PIU (*P*<.001), whereas those in the northern region presented the highest social media identity (*P*<.001). There was no sex difference in PIU (*P*=.10), but male respondents reported greater social media identity (*P*=.04) than their female counterparts (Table S2 of Multimedia Appendix 1).

PIU, loneliness, depression, and anxiety symptoms were positively correlated (*P*<.001), whereas social media identity was negatively correlated with loneliness, depression, and anxiety symptoms (*P*<.001) (Table 2).

**Table 1:** Measurement scores of the mental health conditions, loneliness, problematic internet use, and social media identity of the study participants (N=21,292).

Variables	Mean (SD) score	Median (IQR) score
Depression	6.49 (5.51)	6 (2-9)
Anxiety	4.75 (4.62)	4 (0-7)
Loneliness	1.57 (1.61)	1 (0-3)
Problematic internet use	5.80 (5.46)	11 (0-10)
Social media identity	31.12 (10.99)	37 (24-38)

**Table 2:** Spearman correlations of the measured construct (N=21,292).

Variables				Depression		Anxiety		Loneliness		Problematic internet use		Social media identity
**Depression**												
	*r*		1		0.811		0.628		0.449		–0.125	
	*P* value		—^a^		<.001		<.001		<.001		<.001	
**Anxiety**												
	*r*		0.811		1		0.622		0.431		–0.105	
	*P* value		<.001		—		<.001		<.001		<.001	
**Loneliness**												
	*r*		0.628		0.622		1		0.395		–0.156	
	*P* value		<.001		<.001		—		<.001		<.001	
**Problematic internet use**												
	*r*		0.449		0.431		0.395		1		0.007	
	*P* value		<.001		<.001		<.001		—		.29	
**Social media identity**												
		*r*	–0.125		–0.105		–0.156		0.007		1	
		*P* value	<.001		<.001		<.001		.29		—	

^a^Not applicable.

### Hypothesis Testing

PIU was found to be associated with loneliness (model 1 in Table 3), depression (model 2 in Table 3), and anxiety (model 3 in Table 3). This supports hypotheses 1 and 2. Loneliness played a significant mediating role in these associations, as shown in models 2 and 3 in Table 2. The change in *R*^2^ values confirmed the substantial contribution of loneliness as a mediator in the relationships between PIU and depression (*R*^2^ increased from 0.246 to 0.454) and anxiety (*R*^2^ increased from 0.223 to 0.446).

**Figure 2:**
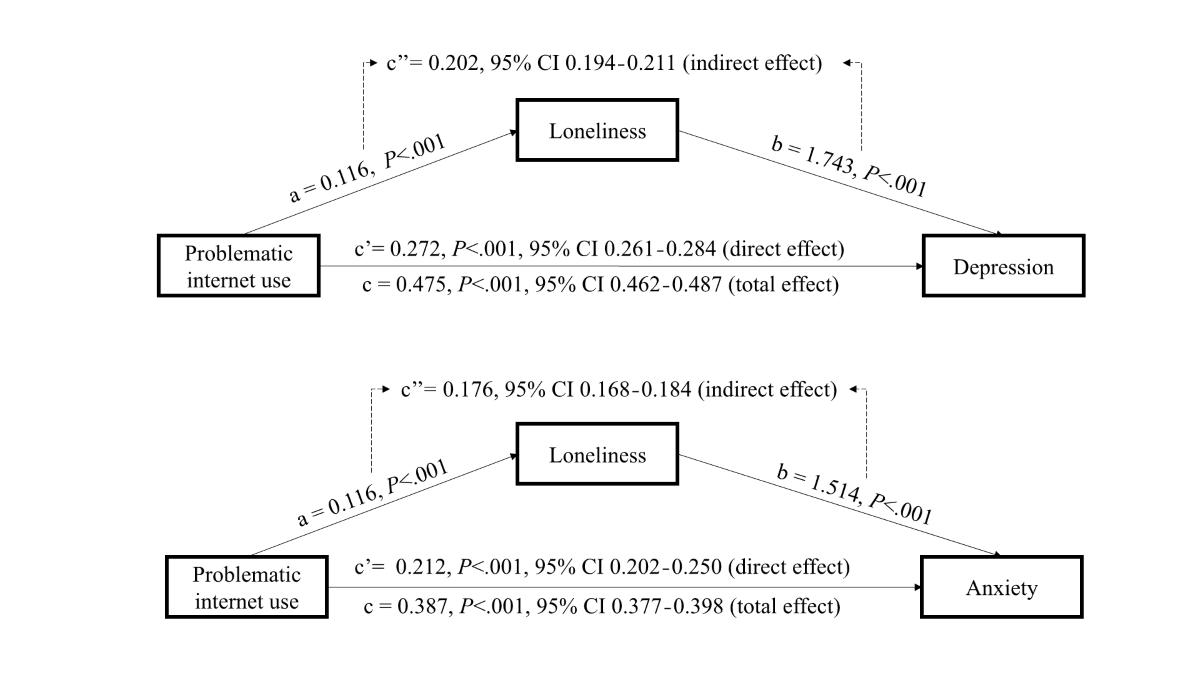
Mediating model of problematic internet use on depression and anxiety via loneliness. "a" represents the coefficient between problematic internet use and loneliness, "b" represents the coefficient between loneliness and depression/anxiety, and "c" represents the total coefficient when combined direct coefficient (c') from problematic internet use and depression/anxiety and indirect coefficient (c") between them via loneliness.

The total effect of PIU on depression was 0.475 (95% CI 0.462-0.487), of which 42.53% (0.202, 95% CI 0.193-0.211) was explained by the indirect effect mediated through loneliness. Similarly, the total effect of PIU on anxiety was 0.387 (95% CI 0.377-0.398), with 45.48% (0.176, 95% CI 0.168-0.184) explained by the indirect effect mediated through loneliness (see Figure 2). This finding supports hypothesis 3.

**Table 3:** Results of the mediating effect of loneliness in the relationship between problematic internet use and mental health.

Predictors	Model 1^a^ (Y=loneliness)	Model 2^b^ (Y=PHQ^c^)	Model 3^d^ (Y=GAD^e^)
PIU^f^, β (95% CI)	0.116 (0.112-0.120)	0.272 (0.261-0.284)	0.212 (0.202-0.222)
Loneliness, β (95% CI)	N/A^g^	1.743 (1.705-1.781)	1.514 (1.482-1.546)

^a^*F*_33, 21,258_=160.675; *R*^2^=0.200.

^b^*F*_34, 21,257_=519.443; *R*^2^=0.454.

^c^PHQ: Patient Health Questionnaire.

^d^*F*_34, 21,257_=502.896; *R*^2^=0.446.

^e^GAD: Generalized Anxiety Disorder scale.

^f^PIU: problematic internet use.

^g^N/A: not applicable.

**Figure 3:**
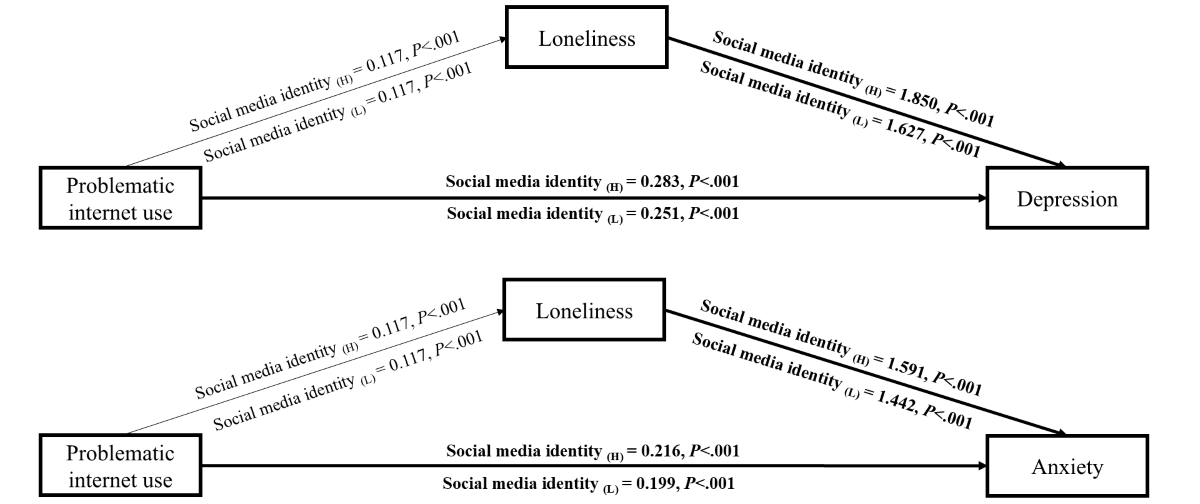
For each pathway, social media identity (H) and social media identity (L) represent the associations among problematic internet use, loneliness, and depression/anxiety when the level of social media identity is set at the mean score plus one standard deviation or minus one standard deviation, respectively. *P* value indicates the significance of the pathway, and bold paths and values indicate significant differences between different levels of social media identity.

Social media identity moderated the effect of PIU on depression (β=0.002, 95% CI 0.001-0.002; model 5 in Table 4) and the effect of loneliness on depression (β=0.010, 95% CI 0.007-0.013; model 5 in Table 4). Similarly, social media identity moderated the effect of PIU on anxiety (β=0.001, 95% CI 0.000-0.002; model 6 in Table 4) and the effect of loneliness on anxiety (β=0.007, 95% CI 0.004-0.010; model 6 in Table 4). Higher levels of social media identity exacerbated the effects of PIU on mental health problems, supporting hypothesis 4. However, the moderating effect of social media identity on the association between PIU and loneliness was statistically insignificant (β=0.000, 95% CI –0.003 to 0.003; model 4 in Table 4), despite the direct association between higher levels of social media identity and lower levels of loneliness (β=–0.018, 95% CI –0.020 to –0.016; model 4 in Table 4). Multimedia Appendix 2 and Figure 3 present the conditional effects of social media identity on the paths between PIU and mental health symptoms.

**Table 4:** Results of the moderated mediation model of social media identity and loneliness in the relationship between problematic internet use and mental health.

Predictors	Model 4^a^ (Y=Loneliness)	Model 5^b^ (Y=PHQ^c^)	Model 6^d^ (Y=GAD^e^)
PIU^f^, β (95% CI)	0.117 (0113 to 0.121)	0.267 (0.255 to 0.279)	0.207 (0.198 to 0.217)
Loneliness, β (95% CI)	N/A^g^	1.739 (1.700 to 1.778)	1.516 (1.484 to 1.549)
Social media identity, β (95% CI)	–0.018 (–0.020 to –0.016)	–0.005 (–0.010 to 0.001)	0.002 (–0.002 to 0.007)
Social media identity × PIU, β (95% CI)	0.000 (–0.003 to 0.003)	0.002 (0.001 to 0.002)	0.001 (0.000 to 0.002)
Social media identity × loneliness, β (95% CI)	N/A	0.010 (0.007 to 0.013)	0.007 (0.004 to 0.010)

^a^*F*_35, 21,256_=165.462; *R*^2^=0.214.

^b^*F*_37, 21,254_=481.285; *R*^2^=0.456.

^c^PHQ: Patient Health Questionnaire.

^d^*F*_37, 21,254_=464.138; *R*^2^=0.447.

^e^GAD: Generalized Anxiety Disorder scale.

^f^PIU: problematic internet use.

^g^N/A: not applicable.

The effect of PIU on depression, including the indirect effect via loneliness, was more significant among respondents with a higher social media identity than among those with lower social media identity. This difference was manifested as a 0.032 variation in the direct effect and a 0.025 difference in the indirect effect, with a bootstrapped 95% CI of 0.005-0.044. Similarly, the effect of PIU on anxiety, including the indirect effect via loneliness, was significantly more pronounced among respondents with higher social media identity than among those with lower social media identity. This difference amounted to a 0.017 variation in the direct effect and a 0.017 difference in the indirect effect, with a bootstrapped 95% CI of 0.000-0.033 (Table 5).

The subgroup analyses based on sex and age confirmed the moderated mediation models tested in this study (Table S4 of Multimedia Appendix 1). In all the subgroups, except for those older than 60 years, the indirect effects of PIU on depression and anxiety via loneliness exceeded 40% of the total effect. Additionally, higher levels of social media identity were associated with greater impacts of PIU on depression and anxiety in nearly all subgroups, except the group aged 50-59 years, where this effect was not observed for the impact of PIU on depression.

Alternative model testing revealed that PIU mediated the effects of loneliness on depression and anxiety (Tables S5 and S6 of Multimedia Appendix 1). However, the indirect effect explained only 16.12% and 14.66% of the total effect of loneliness on depression and anxiety, respectively. The change in *R*^2^ values also indicated a limited contribution of PIU as a mediator.

**Table 5:** Conditional indirect and direct effects of social media identity.

Social media identity			Indirect effect		Boot SE		Boot LLCI-ULCI^a^		Direct effect		LLCI-ULCI
**Depression**											
	Average	0.203		0.005		0.178-0.212		0.267		0.255-0.279	
	Low (mean – 1 SD)	0.190		0.006		0.178-0.203		0.251		0.234-0.268	
	High (mean + 1 SD)	0.216		0.007		0.201-0.229		0.283		0.268-0.298	
	Difference	0.025		0.010		0.005-0.044		0.032		N/A^b^	
**Anxiety**											
	Average	0.177		0.004		0.169-0.185		0.207		0.198-0.217	
	Low (mean – 1 SD)	0.169		0.006		0.158-0.180		0.199		0.185-0.213	
	High (mean + 1 SD)	0.185		0.006		0.174-0.197		0.216		0.204-0.229	
	Difference	0.017		0.008		0.000-0.033		0.017		N/A	

^a^LLCI-ULCI: lower limit of confidence interval to upper limit of confidence interval.

^b^N/A: not applicable.

## Discussion

### Main Findings

This study reveals that increased PIU is linked to increased depression and anxiety symptoms. Loneliness acts as a mediator in this association, accounting for almost half of the total effects. Notably, while a higher social media identity may decrease loneliness, it intensifies the impacts of loneliness and PIU on depression and anxiety.

### Comparisons With the Existing Evidence

The study confirms a significant effect of PIU on loneliness, although the direction of the relationship remains inconclusive. This aligns with the findings of most existing studies [25,70,71], indicating an internet displacement effect, in which the internet replaces offline interactions rather than a stimulation effect, in which the internet enhances existing relationships [25]. Previous studies have extensively documented the positive associations of PIU with depression and anxiety [17-20,40,72], as well as the positive associations of loneliness with depression and anxiety [73-76]. These associations were confirmed in this study.

In our study, we discovered that loneliness serves as a significant mediator in the link between PIU and mental health disorders, explaining nearly half of the total effects on both depression and anxiety symptoms. Notably, loneliness can be alleviated by enhancing social skills and strengthening social support [77], providing a novel approach to mitigate the negative consequences of PIU on mental health [78]. However, the substantial direct effects of PIU on depression and anxiety symptoms remain unclear and warrant further exploration. Existing evidence has suggested that sleep disturbance may be a potential pathway through which PIU affects mental health [79,80]. Consequently, future research is needed to deepen our understanding of the underlying reasons for the association between PIU and mental health.

Social media has produced revolutionary changes in people's daily lives, profoundly impacting their health. Previous studies have established connections between PIU and social media identity [31] as well as between social media identity and loneliness [30,51,52]. This study expands our existing knowledge by exploring the role of social media identity in the effects of PIU on both loneliness and mental health disorders. We found that higher levels of social media identity have the potential to exacerbate the effects of PIU on mental health disorders. Social media identity is formed as individuals seek connections with like-minded others online [31,81], which can reduce feelings of loneliness, as indicated in this study and others [82-84]. However, this reduction in loneliness does not appear to be strong enough to counterbalance the exacerbating effects of social media identity on the association between PIU and mental health disorders. One plausible explanation is that individuals with high levels of social media identity become deeply engrossed in the virtual online space at the expense of offline social interactions [85,86]. This may result in reduced ability to obtain social support from traditional relationships such as family members, friends, and work colleagues. Online relationships are often criticized for being superficial and lacking authenticity, and they cannot fully replace the role of offline relationships [87]. People with a high level of social media identity may feel less lonely but have limited access to offline interactions and social support, thereby increasing their risk of depression and anxiety [31,37].

Although the primary focus of this study was not on the prevalence of depression and anxiety, our study participants appeared to exhibit similar levels of depression and anxiety symptoms as those reported in another national study conducted in 2021 [88]. In our study, approximately 22.80% (4854/21,292) and 14.20% (3023/21,292) of the respondents were identified as experiencing moderate or severe depression and anxiety, respectively [89,90]. Consistent with previous studies conducted during the COVID-19 pandemic [2], our research also identifies the female sex and young age groups such as those aged 18-29 years as significant risk factors for mental health disorders. Recent studies have revealed a rapid increase in PIU, which is the most prevalent among men and younger age groups [91,92]. However, our study suggests that the effects of PIU on mental health disorders are similar between male and female respondents.

### Policy Implications

As loneliness is a significant mediator between PIU and depression and anxiety, it is beneficial to consider a comprehensive strategy that addresses both PIU and loneliness to mitigate the negative mental health outcomes associated with PIU. For educators and caregivers, existing studies have shown that active educational interventions, including alternative activity recordings and internet diary writing, were more likely to decrease internet addiction [93]. Additionally, policymakers can also introduce multilevel approaches that target internet users, online content, and the internet industry to address this challenge [94]. For example, China has issued a series of policies in which gaming companies have been required to impose restrictions, such as limiting young users to 1 hour of play on Fridays and weekends. Furthermore, the awareness and knowledge of the public should also be improved, especially with respect to risky online behaviors [93]. Research indicates that intervention programs focused on specific online risks such as gambling, cyberbullying, and online gaming tend to be more effective than general PIU interventions [95]. Offline strategies that enhance social connectedness and social support have also demonstrated their effectiveness in reducing loneliness [96] and can be incorporated into PIU management efforts. Social media identity plays a pivotal role in amplifying the mental health consequences of PIU. This underscores the importance of helping individuals balance their online and offline relationships. Although online connections can temporarily relieve loneliness, they should not substitute or jeopardize the value of real-world connections. Potential intervention strategies may involve public education campaigns to increase awareness and encourage responsible online behavior, as well as strengthening offline social activities to promote face-to-face interactions and social support.

### Strengths and Limitations

To our knowledge, this is the first study to provide valuable insights into the intricate connections among PIU, loneliness, social media identity, and mental health disorders by using moderated mediation modeling. The sample size was large. This study has several limitations. One significant limitation is the cross-sectional design, which prevents us from verifying the bidirectional relationships between PIU and loneliness as well as between PIU and mental health disorders. Future research could benefit from a longitudinal design to explore the dynamic interplay between PIU, loneliness, and mental health over time. Additionally, although we used a moderated mediation effect model to examine the effects of PIU, loneliness, social media identity and their interaction on mental health, the nature of the cross-sectional design allows us to generate only an association rather than a causal relationship between them. Furthermore, the moderating effects were relatively small in this study, which may be susceptible to changes in other populations or concerning other mental health disorders. Thus, this study should be interpreted with caution. Furthermore, other unexamined factors and pathways may contribute to the relationship between PIU and mental health. For example, variables related to sleep patterns and sedentary lifestyles could play a role in both PIU and mental health disorders [97]. These factors were not included in our study because of data unavailability. Finally, although the mental health status of the participants was assessed on the basis of their experiences during the 2 weeks before the survey, the results were still vulnerable to potential recall bias. The excluded participants were more likely to be men older than 50 years, resulting in a slight underrepresentation of these populations in this study.

### Conclusions

PIU is linked to symptoms of depression and anxiety, and our research underscores the crucial role of loneliness as a mediator in this association. Loneliness is responsible for nearly half of the total effects of PIU on depression and anxiety symptoms. Intriguingly, although social media identity may offer some relief from loneliness, it paradoxically exacerbates the impact of PIU on depression and anxiety. In light of these findings, a comprehensive and multifaceted approach to PIU management, encompassing both online and offline interventions, is warranted.

## Appendix

Multimedia Appendix 1 Supplementary data.

Multimedia Appendix 2 Moderating effects for the all paths of the direct and indirect effects of the mediational model. A: Moderating effect of social media identity on the effect of problematic internet use on loneliness; B: Moderating effect of social media identity on the effect of loneliness on depression; C: Moderating effect of social media identity on the effect of loneliness on anxiety; D: Moderating effect of social media identity on the effect of problematic internet use on depression; E: Moderating effect of social media identity on the effect of problematic internet use on anxiety.

## Abbreviations

GAD-7: 7-item Generalized Anxiety Disorder scale
PBICR: Psychology and Behavior Investigation of Chinese Residents
PHQ-9: 9-item Patient Health Questionnaire
PIU: problematic internet use

## Data Availability

The corresponding author will make the datasets used and analyzed in this study available upon reasonable request.
