# Supplementary material for: The Moderated Mediating Effects of Social Media Identity and Loneliness on the Relationship Between Problematic Internet Use and Mental Health in China: Nationwide Cross-Sectional Questionnaire Study
Source: J Med Internet Res. 2025 Feb 26;27:e57907. doi: 10.2196/57907 (PMC11904383; doi:10.2196/57907)
Supplement: Multimedia Appendix 1 [file jmir_v27i1e57907_app1.docx]

**Supplementary Tables S1-S6**

| **Table S1 Demographics of participants** | | |
| --- | --- | --- |
| **Characteristics** | **Number (N)** | **Percentage (%)** |
| Sex |  |  |
| Male | 10596 | 49.77% |
| Female | 10696 | 50.23% |
| Age group |  |  |
| <18 | 2103 | 9.88% |
| >=18 & <30 | 6195 | 29.10% |
| >=30 & <40 | 2951 | 13.86% |
| >=40 & <50 | 3572 | 16.78% |
| >=50 & <60 | 2614 | 12.28% |
| >=60 & <70 | 2215 | 10.40% |
| >=70 | 1642 | 7.71% |
| Educational level |  |  |
| No formal education | 1064 | 5.00% |
| Primary school | 2122 | 9.97% |
| Junior high school | 3455 | 16.23% |
| Senior high school/Technical secondary school | 5080 | 23.86% |
| University/Junior college | 8759 | 41.14% |
| Postgraduate | 812 | 3.81% |
| Employment |  |  |
| Full-time students | 6453 | 30.31% |
| Employed | 7445 | 34.97% |
| Freelancer | 2548 | 11.97% |
| Retirement | 2658 | 12.48% |
| Unemployed | 2188 | 10.28% |
| Average family income (per person) |  |  |
| <=1000 | 1242 | 5.83% |
| >1000 & <=2000 | 2388 | 11.22% |
| >2000 & <=3000 | 3270 | 15.36% |
| >3000 & <=4000 | 3598 | 16.90% |
| >4000 & <=5000 | 2920 | 13.71% |
| >5000 & <=6000 | 2332 | 10.95% |
| >6000 | 5542 | 26.03% |
| Marital status |  |  |
| Unmarried | 8336 | 39.15% |
| Married | 12053 | 56.61% |
| Divorce/widowed | 903 | 4.24% |
| Living area |  |  |
| Rural | 6462 | 30.35% |
| Urban | 14830 | 69.65% |
| Living Regions |  |  |
| North | 4398 | 20.66% |
| Northeast | 1336 | 6.27% |
| East | 4213 | 19.79% |
| Central | 1989 | 9.34% |
| South | 2635 | 12.38% |
| Southwest | 2978 | 13.99% |
| Northwest | 3743 | 17.58% |
| Chronic disease |  |  |
| Yes | 5432 | 25.51% |
| No | 15860 | 74.49% |

| **Table S2 Differences of variables between participants with various demographics** | | | | | | | | | | |
| --- | --- | --- | --- | --- | --- | --- | --- | --- | --- | --- |
| **Characteristics** | **Loneliness** | **p value** | **PIU** | **p value** | **Social Media Identity** | **p value** | **Depression** | **p value** | **Anxiety** | **p value** |
| Sex |  | <0.001 |  | 0.101 |  | 0.040 |  | 0.068 |  | 0.003 |
| Male | 1.519 (1.613) |  | 5.913 (5.622) |  | 31.235 (11.310) |  | 6.523 (5.765) |  | 4.728 (4.737) |  |
| Female | 1.623 (1.606) |  | 5.678 (5.294) |  | 31.005 (10.668) |  | 6.456 (5.238) |  | 4.780 (4.502) |  |
| Age group |  | <0.001 |  | <0.001 |  | <0.001 |  | <0.001 |  | <0.001 |
| <18 | 1.673 (1.796) |  | 7.556 (5.805) |  | 31.646 (11.474) |  | 6.859 (6.427) |  | 5.014 (5.319) |  |
| >=18 & <30 | 1.859 (1.684) |  | 7.896 (5.362) |  | 32.318 (10.031) |  | 7.230 (5.628) |  | 5.302 (4.865) |  |
| >=30 & <40 | 1.627 (1.590) |  | 6.075 (5.352) |  | 31.259 (10.897) |  | 6.796 (5.551) |  | 5.066 (4.693) |  |
| >=40 & <50 | 1.384 (1.490) |  | 4.901 (4.897) |  | 30.521 (11.055) |  | 6.139 (5.077) |  | 4.392 (4.253) |  |
| >=50 & <60 | 1.209 (1.444) |  | 4.067 (4.757) |  | 29.909 (11.983) |  | 5.484 (5.125) |  | 4.017 (4.208) |  |
| >=60 & <70 | 1.360 (1.490) |  | 3.530 (4.904) |  | 31.373 (10.522) |  | 5.727 (5.264) |  | 4.262 (4.218) |  |
| >=70 | 1.525 (1.560) |  | 2.864 (4.506) |  | 28.558 (12.101) |  | 6.065 (5.000) |  | 4.420 (4.156) |  |
| Educational level |  | <0.001 |  | <0.001 |  | <0.001 |  | <0.001 |  | <0.001 |
| No formal education | 1.490 (1.551) |  | 3.367 (4.973) |  | 28.648 (12.115) |  | 6.262 (5.320) |  | 4.724 (4.358) |  |
| Primary school | 1.316 (1.479) |  | 3.639 (4.752) |  | 29.667 (11.917) |  | 5.649 (5.016) |  | 4.162 (4.108) |  |
| Junior high school | 1.266 (1.531) |  | 4.741 (5.228) |  | 30.060 (11.420) |  | 5.679 (5.418) |  | 4.159 (4.467) |  |
| Senior high school/Technical secondary school | 1.649 (1.673) |  | 6.344 (5.598) |  | 31.316 (10.745) |  | 6.681 (5.628) |  | 4.874 (4.738) |  |
| University/Junior college | 1.714 (1.611) |  | 6.598 (5.380) |  | 31.851 (10.510) |  | 6.883 (5.479) |  | 5.022 (4.674) |  |
| Postgraduate | 1.619 (1.675) |  | 7.010 (5.600) |  | 33.537 (10.355) |  | 6.995 (6.226) |  | 5.245 (5.140) |  |
| Employment |  | <0.001 |  | <0.001 |  | <0.001 |  | <0.001 |  | <0.001 |
| Full-time student | 1.870 (1.725) |  | 8.140 (5.448) |  | 32.261 (10.469) |  | 7.211 (5.849) |  | 5.233 (5.021) |  |
| Employed | 1.446 (1.539) |  | 5.507 (5.193) |  | 31.324 (10.923) |  | 6.241 (5.311) |  | 4.600 (4.462) |  |
| Freelancer | 1.365 (1.530) |  | 4.539 (5.010) |  | 30.094 (11.347) |  | 6.168 (5.376) |  | 4.535 (4.465) |  |
| Retirement | 1.412 (1.499) |  | 3.666 (4.817) |  | 30.842 (11.409) |  | 5.859 (5.126) |  | 4.275 (4.189) |  |
| Unemployed | 1.551 (1.596) |  | 3.910 (5.113) |  | 28.586 (11.278) |  | 6.349 (5.506) |  | 4.707 (4.484) |  |
| Average family income (per person) | | <0.001 |  | <0.001 |  | <0.001 |  | <0.001 |  | <0.001 |
| <=1000 | 1.995 (1.725) |  | 5.714 (5.947) |  | 27.192 (11.899) |  | 8.033 (6.301) |  | 5.861 (5.243) |  |
| >1000 & <=2000 | 1.536 (1.604) |  | 5.292 (5.391) |  | 28.420 (11.677) |  | 6.290 (5.343) |  | 4.550 (4.509) |  |
| >2000 & <=3000 | 1.546 (1.580) |  | 5.423 (5.258) |  | 29.786 (11.090) |  | 6.458 (5.374) |  | 4.705 (4.519) |  |
| >3000 & <=4000 | 1.445 (1.567) |  | 5.192 (5.247) |  | 31.442 (10.428) |  | 5.897 (5.252) |  | 4.327 (4.312) |  |
| >4000 & <=5000 | 1.548 (1.568) |  | 5.827 (5.325) |  | 31.496 (10.796) |  | 6.348 (5.277) |  | 4.669 (4.420) |  |
| >5000 & <=6000 | 1.509 (1.580) |  | 5.983 (5.222) |  | 31.884 (10.333) |  | 6.331 (5.387) |  | 4.587 (4.538) |  |
| >6000 | 1.622 (1.649) |  | 6.546 (5.705) |  | 33.219 (10.582) |  | 6.774 (5.711) |  | 5.017 (4.853) |  |
| Marital status |  | <0.001 |  | <0.001 |  | <0.001 |  | <0.001 |  | <0.001 |
| Unmarried | 1.863 (1.717) |  | 7.950 (5.497) |  | 32.024 (10.566) |  | 7.318 (5.885) |  | 5.331 (5.030) |  |
| Married | 1.339 (1.485) |  | 4.448 (4.948) |  | 30.756 (11.010) |  | 5.869 (5.138) |  | 4.335 (4.263) |  |
| Divorce/widowed | 1.981 (1.672) |  | 3.891 (5.176) |  | 27.609 (12.363) |  | 7.128 (5.575) |  | 5.027 (4.682) |  |
| Living area |  | 0.013 |  | 0.009 |  | <0.001 |  | <0.001 |  | <0.001 |
| Rural | 1.611 (1.616) |  | 5.688 (5.516) |  | 30.411 (10.777) |  | 6.739 (5.564) |  | 4.933 (4.609) |  |
| Urban | 1.554 (1.607) |  | 5.842 (5.436) |  | 31.428 (11.072) |  | 6.381 (5.478) |  | 4.677 (4.624) |  |
| Living Regions |  | <0.001 |  | <0.001 |  | <0.001 |  | <0.001 |  | <0.001 |
| North | 1.465 (1.644) |  | 5.937 (5.706) |  | 32.352 (10.983) |  | 6.320 (5.830) |  | 4.582 (4.813) |  |
| Northeast | 1.844 (1.610) |  | 6.966 (5.717) |  | 31.321 (10.893) |  | 7.622 (5.862) |  | 5.686 (5.012) |  |
| East | 1.558 (1.577) |  | 5.635 (5.260) |  | 30.241 (11.629) |  | 6.283 (5.214) |  | 4.589 (4.426) |  |
| Central | 1.772 (1.656) |  | 6.099 (5.588) |  | 29.631 (11.666) |  | 7.011 (5.378) |  | 4.957 (4.642) |  |
| South | 1.513 (1.589) |  | 5.280 (5.276) |  | 31.719 (10.711) |  | 6.122 (5.583) |  | 4.572 (4.743) |  |
| Southwest | 1.757 (1.641) |  | 5.871 (5.430) |  | 31.186 (10.278) |  | 6.805 (5.386) |  | 4.942 (4.588) |  |
| Northwest | 1.402 (1.536) |  | 5.533 (5.303) |  | 30.903 (10.489) |  | 6.248 (5.332) |  | 4.683 (4.332) |  |
| Chronic disease |  | <0.001 |  | <0.001 |  | <0.001 |  | <0.001 |  | <0.001 |
| Yes | 1.767 (1.635) |  | 5.064 (5.484) |  | 29.926 (11.268) |  | 7.282 (5.525) |  | 5.392 (4.729) |  |
| No | 1.504 (1.596) |  | 6.050 (5.431) |  | 31.528 (10.867) |  | 6.218 (5.474) |  | 4.536 (4.563) |  |

| **Table S3 The moderated mediation model regarding depression** | | | | | | | | | | | | |
| --- | --- | --- | --- | --- | --- | --- | --- | --- | --- | --- | --- | --- |
|  | **Mediation model** | | | | | | **Moderated mediation model** | | | | | |
| **Predictors** | **Model 1 (Y=Loneliness)** | | **Model 2 (Y=Depression)** | | **Model 3 (Y=Anxiety)** | | **Model 4 (Y=Loneliness)** | | **Model 5 (Y=Depression)** | | **Model 6 (Y=Anxiety)** | |
|  | **β** | **95%CI** | **β** | **95%CI** | **β** | **95%CI** | **β** | **95%CI** | **β** | **95%CI** | **β** | **95%CI** |
| PIU | 0.116 | (0.112, 0.120) | 0.272 | (0.261, 0.284) | 0.212 | (0.202, 0.222) | 0.117 | (0113, 0.121) | 0.267 | (0.255, 0.279) | 0.207 | (0.198, 0.217) |
| Loneliness |  |  | 1.743 | (1.705, 1.781) | 1.514 | (1.482, 1.546) |  |  | 1.739 | (1.700, 1.778) | 1.516 | (1.484, 1.549) |
| Social media identity |  |  |  |  |  |  | -0.018 | (-0.020, -0.016) | -0.005 | (-0.010, 0.001) | 0.002 | (-0.002, 0.007) |
| Social media identity × PIU |  |  |  |  |  |  | 0.000 | (-0.003, 0.003) | 0.002 | (0.001, 0.002) | 0.001 | (0.000, 0.002) |
| Social media identity ×loneliness |  |  |  |  |  |  |  |  | 0.010 | (0.007, 0.013) | 0.007 | (0.004, 0.010) |
| F | 160.675 | | 519.443 | | 502.896 | | 165.462 | | 481.285 | | 464.138 | |
| R^2^ | 0.200 | | 0.454 | | 0.446 | | 0.214 | | 0.456 | | 0.447 | |

| **Table S4 The moderated mediation model regarding depression** | | | | | | | | | | | | |
| --- | --- | --- | --- | --- | --- | --- | --- | --- | --- | --- | --- | --- |
| **Predictors** | **Model 1 (Y=Loneliness)** | | **Model 2 (Y=Depression)** | | **Model 3 (Y=Anxiety)** | | **Model 4 (Y=Loneliness)** | | **Model 5 (Y=Depression)** | | **Model 6 (Y=Anxiety)** | |
|  | **β** | **95%CI** | **β** | **95%CI** | **β** | **95%CI** | **β** | **95%CI** | **β** | **95%CI** | **β** | **95%CI** |
| **Male** |  |  |  |  |  |  |  |  |  |  |  |  |
| PIU | 0.116 | (0.111, 0.121) | 0.271 | (0.255, 0.288) | 0.209 | (0.196, 0.223) | 0.116 | (0.111, 0.121) | 0.261 | (0.245, 0.278) | 0.202 | (0.189, 0.216) |
| Loneliness |  |  | 1.862 | (1.806, 1.917) | 1.590 | (1.544, 1.635) |  |  | 1.858 | (1.802, 1.913) | 1.592 | (1.546, 1.638) |
| Social media identity |  |  |  |  |  |  | -0.016 | (-0.019, -0.014) | 0.003 | (-0.004, 0.011) | 0.008 | (0.001, 0.014) |
| Social media identity × PIU |  |  |  |  |  |  | 0.000 | (-0.000, 0.001) | 0.002 | (0.001, 0.004) | 0.001 | (0.000, 0.002) |
| Social media identity × Loneliness |  |  |  |  |  |  |  |  | 0.013 | (0.009, 0.018) | 0.010 | (0.006, 0.013) |
| F | 91.094 | | 294.754 | | 291.612 | | 92.263 | | 274.240 | | 270.016 | |
| R^2^ | 0.216 | | 0.479 | | 0.477 | | 0.229 | | 0.483 | | 0.479 | |
| **Female** |  |  |  |  |  |  |  |  |  |  |  |  |
| PIU | 0.117 | (0.111, 0.122) | 0.270 | (0.253, 0.286) | 0.211 | (0.197, 0.226) | 0.118 | (0.112, 0.123) | 0.270 | (0.253, 0.287) | 0.211 | (0.196, 0.225) |
| Loneliness |  |  | 1.625 | (1.573, 1.677) | 1.438 | (1.392, 1.483) |  |  | 1.612 | (1.560, 1.665) | 1.435 | (1.389, 1.481) |
| Social media identity |  |  |  |  |  |  | -0.020 | (-0.023, -0.018) | -0.015 | (-0.022, -0.007) | (0.004) | (-0.010, 0.003) |
| Social media identity × PIU |  |  |  |  |  |  | -0.001 | (-0.001, 0.000) | 0.000 | (-0.001, 0.002) | 0.000 | (-0.001, 0.001) |
| Social media identity × Loneliness |  |  |  |  |  |  |  |  | 0.006 | (0.001, 0.010) | 0.003 | (-0.001 0.007) |
| F | 76.814 | | 243.047 | | 230.224 | | 80.493 | | 223.891 | | 211.202 | |
| R^2^ | 0.187 | | 0.429 | | 0.416 | | 0.204 | | 0.431 | | 0.416 | |
| **Age: 12-17 (Not adjust for education & employment & married status)** | | | |  |  |  |  |  |  |  |  |  |
| PIU | 0.120 | (0.108, 0.131) | 0.212 | (0.175, 0.250) | 0.170 | (0.140, 0.201) | 0.122 | (0.111, 0.134) | 0.213 | (0.176, 0.251) | 0.168 | (0.137, 0.199) |
| Loneliness |  |  | 2.007 | (1.881, 2.133) | 1.741 | (1.637, 1.845) |  |  | 1.994 | (1.867, 2.121) | 1.739 | (1.635, 1.844) |
| Social media identity |  |  |  |  |  |  | -0.019 | (-0.025, -0.014) | -0.009 | (-0.026, 0.001) | 0.000 | (-0.014, 0.015) |
| Social media identity × PIU |  |  |  |  |  |  | 0.000 | (-0.001, 0.001) | -0.001 | (-0.004, 0.002) | 0.001 | (-0.002, 0.003) |
| Social media identity × Loneliness |  |  |  |  |  |  |  |  | 0.015 | (0.006, 0.025) | 0.006 | (-0.002, 0.014) |
| F | 38.312 | | 101.587 | | 103.280 | | 37.477 | | 90.188 | | 91.070 | |
| R^2^ | 0.279 | | 0.518 | | 0.522 | | 0.293 | | 0.521 | | 0.523 | |
| **Age: 18-29 (Not adjust for employment & married status)** | | |  |  |  |  |  |  |  |  |  |  |
| PIU | 0.117 | (0.110, 0.124) | 0.268 | (0.247, 0.290) | 0.206 | (0.187, 0.225) | 0.117 | (0.109, 0.124) | 0.262 | (0.241, 0.284) | 0.204 | (0.185, 0.223) |
| Loneliness |  |  | 1.634 | (1.565, 1.704) | 1.450 | (1.390, 1.511) |  |  | 1.628 | (1.559, 1.698) | 1.446 | (1.385, 1.507) |
| Social media identity |  |  |  |  |  |  | -0.024 | (-0.027, -0.020) | -0.004 | (-0.015, 0.007) | (0.004) | (-0.013, 0.006) |
| Social media identity × PIU |  |  |  |  |  |  | 0.000 | (-0.001, 0.001) | 0.004 | (0.002, 0.006) | 0.002 | (0.000, 0.003) |
| Social media identity × Loneliness |  |  |  |  |  |  |  |  | 0.000 | (-0.006, 0.006) | (0.000) | (-0.006, 0.005) |
| F | 61.482 | | 213.255 | | 200.838 | | 63.718 | | 189.036 | | 177.039 | |
| R^2^ | 0.173 | | 0.432 | | 0.417 | | 0.192 | | 0.434 | | 0.418 | |
| **Age: 30-39** |  |  |  |  |  |  |  |  |  |  |  |  |
| PIU | 0.112 | (0.102, 0.122) | 0.223 | (0.193, 0.253) | 0.168 | (0.142, 0.194) | 0.110 | (0.100, 0.120) | 0.218 | (0.188, 0.248) | 0.164 | (0.138, 0.190) |
| Loneliness |  |  | 1.929 | (1.829, 2.029) | 1.653 | (1.565, 1.740) |  |  | 1.915 | (1.814, 2.016) | 1.646 | (1.558, 1.734) |
| Social media identity |  |  |  |  |  |  | -0.017 | (-0.022, -0.013) | -0.008 | (-0.022, 0.006) | (0.001) | (-0.013, 0.011) |
| Social media identity × PIU |  |  |  |  |  |  | 0.001 | (-0.000, 0.002) | 0.002 | (-0.000, 0.005) | 0.002 | (0.000, 0.005) |
| Social media identity × Loneliness |  |  |  |  |  |  |  |  | 0.005 | (-0.004, 0.013) | (0.001) | (-0.008, 0.007) |
| F | 27.281 | | 105.058 | | 92.094 | | 27.873 | | 95.421 | | 83.412 | |
| R^2^ | 0.201 | | 0.502 | | 0.469 | | 0.217 | | 0.503 | | 0.470 | |
| **Age: 40-49** |  |  |  |  |  |  |  |  |  |  |  |  |
| PIU | 0.120 | (0.111, 0.129) | 0.239 | (0.210, 0.268) | 0.193 | (0.169, 0.218) | 0.120 | (0.110, 0.129) | 0.228 | (0.199, 0.257) | 0.183 | (0.159, 0.208) |
| Loneliness |  |  | 1.700 | (1.606, 1.795) | 1.445 | (1.365, 1.525) |  |  | 1.698 | (1.603, 1.793) | 1.465 | (1.384, 1.515) |
| Social media identity |  |  |  |  |  |  | -0.019 | (-0.023, -0.015) | -0.004 | (-0.016, 0.008) | 0.013 | (0.003, 0.023) |
| Social media identity × PIU |  |  |  |  |  |  | 0.000 | (-0.000, 0.001) | 0.004 | (0.002, 0.007) | 0.003 | (0.001, 0.005) |
| Social media identity × Loneliness |  |  |  |  |  |  |  |  | 0.018 | (0.010, 0.026) | 0.014 | (0.007, 0.020) |
| F | 33.317 | | 96.795 | | 93.060 | | 34.907 | | 90.116 | | 86.114 | |
| R^2^ | 0.202 | | 0.433 | | 0.424 | | 0.222 | | 0.441 | | 0.430 | |
| **Age: 50-59** |  |  |  |  |  |  |  |  |  |  |  |  |
| PIU | 0.113 | (0.103, 0.124) | 0.287 | (0.252, 0.321) | 0.210 | (0.182, 0.239) | 0.113 | (0.102, 0.124) | 0.286 | (0.251, 0.321) | 0.206 | (0.177, 0.234) |
| Loneliness |  |  | 1.730 | (1.616, 1.845) | 1.522 | (1.428, 1.616) |  |  | 1.738 | (1.623, 1.853) | 1.532 | (1.438, 1.626) |
| Social media identity |  |  |  |  |  |  | -0.009 | (-0.014, -0.005) | 0.002 | (-0.012, 0.015) | 0.009 | (-0.002, 0.020) |
| Social media identity × PIU |  |  |  |  |  |  | 0.000 | (-0.001, 0.001) | -0.003 | (-0.006, 0.000) | (0.001) | (-0.003, 0.002) |
| Social media identity × Loneliness |  |  |  |  |  |  |  |  | 0.014 | (0.004, 0.023) | 0.014 | (0.006, 0.022) |
| F | 23.262 | | 71.718 | | 72.318 | | 22.434 | | 65.220 | | 66.122 | |
| R^2^ | 0.195 | | 0.437 | | 0.439 | | 0.201 | | 0.439 | | 0.443 | |
| **Age: 60-69** |  |  |  |  |  |  |  |  |  |  |  |  |
| PIU | 0.110 | (0.098, 0.122) | 0.357 | (0.321, 0.393) | 0.267 | (0.238, 0.295) | 0.112 | (0.100, 0.124) | 0.337 | (0.300, 0.374) | 0.255 | (0.226, 0.284) |
| Loneliness |  |  | 1.730 | (1.612, 1.849) | 1.504 | (1.412, 1.597) |  |  | 1.739 | (1.620, 1.857) | 1.516 | (1.423, 1.610) |
| Social media identity |  |  |  |  |  |  | -0.018 | (-0.024, -0.012) | -0.002 | (-0.018, 0.015) | 0.002 | (-0.011, 0.014) |
| Social media identity × PIU |  |  |  |  |  |  | 0.000 | (-0.001, 0.001) | 0.005 | (0.001, 0.009) | 0.002 | (-0.001, 0.005) |
| Social media identity × Loneliness |  |  |  |  |  |  |  |  | 0.025 | (0.014, 0.036) | 0.019 | (0.011, 0.028) |
| F | 18.876 | | 73.021 | | 80.171 | | 19.266 | | 68.690 | | 74.204 | |
| R^2^ | 0.189 | | 0.483 | | 0.507 | | 0.204 | | 0.494 | | 0.513 | |
| **Age: >=70** |  |  |  |  |  |  |  |  |  |  |  |  |
| PIU | 0.102 | (0.085, 0.119) | 0.336 | (0.289, 0.383) | 0.282 | (0.243, 0.321) | 0.098 | (0.080, 0.116) | 0.347 | (0.297, 0.396) | 0.286 | (0.244, 0.327) |
| Loneliness |  |  | 1.381 | (1.251, 1.511) | 1.176 | (1.068, 1.285) |  |  | 1.364 | (1.233, 1.495) | 1.166 | (1.057, 1.275) |
| Social media identity |  |  |  |  |  |  | -0.012 | (-0.018, -0.005) | -0.028 | (-0.045, -0.011) | (0.014) | (-0.028, 0.001) |
| Social media identity × PIU |  |  |  |  |  |  | 0.002 | (0.001, 0.004) | -0.001 | (-0.005, 0.002) | (0.000) | (-0.003, 0.003) |
| Social media identity × Loneliness |  |  |  |  |  |  |  |  | 0.007 | (-0.004, 0.017) | 0.003 | (-0.006, 0.011) |
| F | 9.937 | | 40.068 | | 39.034 | | 10.287 | | 36.780 | | 35.410 | |
| R^2^ | 0.143 | | 0.410 | | 0.404 | | 0.156 | | 0.415 | | 0.405 | |

| **Table S5 The mediation model of loneliness on depression and anxiety via PIU** | | | | | | | | | | |
| --- | --- | --- | --- | --- | --- | --- | --- | --- | --- | --- |
| **Predictors** | **Model 1 (Y=PIU)** | | **Model 2 (Y=Depression)** | | **Model 3 (Y=Anxiety)** | | **Model 4 (Y=Depression)** | | **Model 5 (Y=Anxiety)** | |
|  | **β** | **95%CI** | **β** | **95%CI** | **β** | **95%CI** | **β** | **95%CI** | **β** | **95%CI** |
| Loneliness | 1.230 | (1.189, 1.270) | 2.078 | (2.041, 2.115) | 1.774 | (1.743, 1.805) | 1.743 | (1.705, 1.781) | 1.514 | (1.482, 1.546) |
| PIU |  |  |  |  |  |  | 0.272 | (0.261, 0.284) | 0.212 | (0.202, 0.222) |
| F | 229.545 | | 429.487 | | 428.869 | | 519.443 | | 502.896 | |
| R^2^ | 0.263 | | 0.400 | | 0.400 | | 0.454 | | 0.446 | |

| **Table S6 Total, direct and indirect effect based on mediation model of loneliness on depression and anxiety via PIU** | | | | | | | | |
| --- | --- | --- | --- | --- | --- | --- | --- | --- |
| **Total, direct and indirect effect** | **Depression** | | | | **Anxiety** | | | |
|  | **Effect size** | **SE** | **LLCI** | **ULCI** | **Effect size** | **SE** | **LLCI** | **ULCI** |
| Total effect | 2.078 | 0.188 | 2.041 | 2.115 | 1.774 | 0.016 | 1.743 | 1.805 |
| Direct effect | 1.743 | 0.194 | 1.705 | 1.781 | 1.514 | 0.016 | 1.482 | 1.546 |
| Indirect effect | 0.335 | 0.012 | 0.311 | 0.358 | 0.260 | 0.010 | 0.242 | 0.280 |
