# Supplementary figures and images for: The Moderated Mediating Effects of Social Media Identity and Loneliness on the Relationship Between Problematic Internet Use and Mental Health in China: Nationwide Cross-Sectional Questionnaire Study
Source: J Med Internet Res. 2025 Feb 26;27:e57907. doi: 10.2196/57907 (PMC11904383; doi:10.2196/57907)

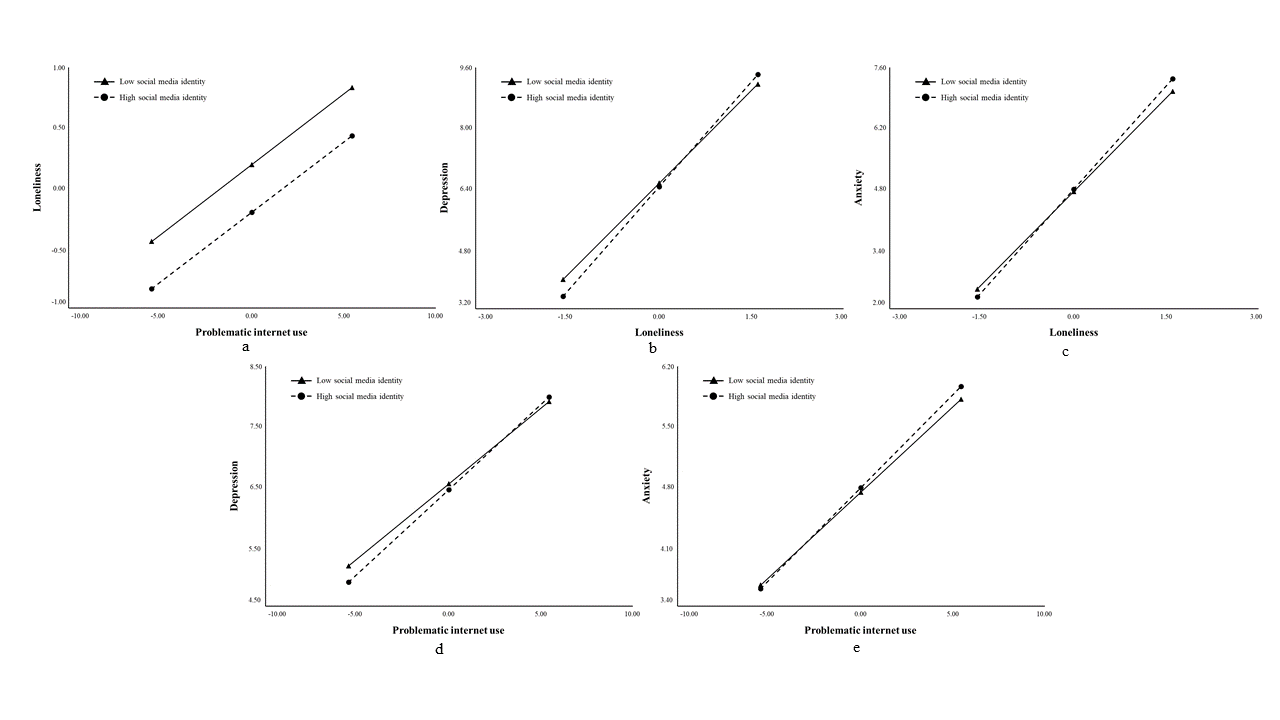

Supplement: Multimedia Appendix 2 [file jmir_v27i1e57907_app2.png]
